# Supplementary material for: Corticosteroids for the Treatment of Internal Temporomandibular Joint Disorders: A Systematic Review and Network Meta-Analysis
Source: J Clin Med. 2024 Aug 4;13(15):4557. doi: 10.3390/jcm13154557 (PMC11313177; doi:10.3390/jcm13154557)
Supplement: Supplementary file 1 [file jcm-13-04557-s001.zip › jcm-3088584-supplementary.pdf]

SUPPLEMENTAL MATERIAL

Supplement 1: PRISMA checklist.

Section/Topic	Item #	Checklist Item	Reported on Page #
**TITLE**			
Title	1	Identify the report as a systematic review *incorporating a network meta-analysis (or related form of meta-analysis).*	1
**ABSTRACT**			
Structured summary	2	Provide a structured summary including, as applicable:<br>**Background:** main objectives<br>**Methods:** data sources; study eligibility criteria, participants, and interventions; study appraisal; and *synthesis methods, such as network meta-analysis.*<br>**Results:** number of studies and participants identified; summary estimates with corresponding confidence/credible intervals; *treatment rankings may also be discussed. Authors may choose to summarize pairwise comparisons against a chosen treatment included in their analyses for brevity.*<br>**Discussion/Conclusions:** limitations; conclusions and implications of findings.<br>**Other:** primary source of funding; systematic review registration number with registry name.	2
**INTRODUCTION**			
Rationale	3	Describe the rationale for the review in the context of what is already known*, including mention of why a network meta-analysis has been conducted.*	3
Objectives	4	Provide an explicit statement of questions being addressed, with reference to participants, interventions, comparisons, outcomes, and study design (PICOS).	3
**METHODS**			
Protocol and registration	5	Indicate whether a review protocol exists and if and where it can be accessed (e.g., Web address); and, if available, provide registration information, including registration number.	4
Eligibility criteria	6	Specify study characteristics (e.g., PICOS, length of follow-up) and report characteristics (e.g., years considered, language, publication status) used as criteria for eligibility, giving rationale. *Clearly describe eligible treatments included in the treatment network, and note whether any have been clustered or merged into the same node (with justification).*	4
Information sources	7	Describe all information sources (e.g., databases with dates of coverage, contact with study authors to identify additional studies) in the search and date last searched.	4
Search	8	Present full electronic search strategy for at least one database, including any limits used, such that it could be repeated.	Supplement 2

Study selection	9	State the process for selecting studies (i.e., screening, eligibility, included in systematic review, and, if applicable, included in the meta-analysis).	4
Data collection process	10	Describe method of data extraction from reports (e.g., piloted forms, independently, in duplicate) and any processes for obtaining and confirming data from investigators.	4-5
Data items	11	List and define all variables for which data were sought (e.g., PICOS, funding sources) and any assumptions and simplifications made.	4-5
**Geometry of the network**	**S1**	Describe methods used to explore the geometry of the treatment network under study and potential biases related to it. This should include how the evidence base has been graphically summarized for presentation, and what characteristics were compiled and used to describe the evidence base to readers.	Supplement 6
Risk of bias within individual studies	12	Describe methods used for assessing risk of bias of individual studies (including specification of whether this was done at the study or outcome level), and how this information is to be used in any data synthesis.	5
Summary measures	13	State the principal summary measures (e.g., risk ratio, difference in means). *Also describe the use of additional summary measures assessed, such as treatment rankings and surface under the cumulative ranking curve (SUCRA) values, as well as modified approaches used to present summary findings from meta-analyses.*	5-6
Planned methods of analysis	14	Describe the methods of handling data and combining results of studies for each network meta-analysis. This should include, but not be limited to:<br>• *Handling of multi-arm trials;*<br>• *Selection of variance structure;*<br>• *Selection of prior distributions in Bayesian analyses; and*<br>• *Assessment of model fit.*	5-6
**Assessment of Inconsistency**	**S2**	Describe the statistical methods used to evaluate the agreement of direct and indirect evidence in the treatment network(s) studied. Describe efforts taken to address its presence when found.	5-6
Risk of bias across studies	15	Specify any assessment of risk of bias that may affect the cumulative evidence (e.g., publication bias, selective reporting within studies).	6
Additional analyses	16	Describe methods of additional analyses if done, indicating which were pre-specified. This may include, but not be limited to, the following:<br>• Sensitivity or subgroup analyses;<br>• Meta-regression analyses;<br>• *Alternative formulations of the treatment network; and*<br>• *Use of alternative prior distributions for Bayesian analyses (if applicable).*	6

**RESULTS†**			
Study selection	17	Give numbers of studies screened, assessed for eligibility, and included in the review, with reasons for exclusions at each stage, ideally with a flow diagram.	7
**Presentation of network structure**	**S3**	Provide a network graph of the included studies to enable visualization of the geometry of the treatment network.	Supplement 6
**Summary of network geometry**	**S4**	Provide a brief overview of characteristics of the treatment network. This may include commentary on the abundance of trials and randomized patients for the different interventions and pairwise comparisons in the network, gaps of evidence in the treatment network, and potential biases reflected by the network structure.	8
Study characteristics	18	For each study, present characteristics for which data were extracted (e.g., study size, PICOS, follow-up period) and provide the citations.	7
Risk of bias within studies	19	Present data on risk of bias of each study and, if available, any outcome level assessment.	7
Results of individual studies	20	For all outcomes considered (benefits or harms), present, for each study: 1) simple summary data for each intervention group, and 2) effect estimates and confidence intervals. *Modified approaches may be needed to deal with information from larger networks.*	7-10
Synthesis of results	21	Present results of each meta-analysis done, including confidence/credible intervals. *In larger networks, authors may focus on comparisons versus a particular comparator (e.g. placebo or standard care), with full findings presented in an appendix. League tables and forest plots may be considered to summarize pairwise comparisons.* If additional summary measures were explored (such as treatment rankings), these should also be presented.	7-10
**Exploration for inconsistency**	**S5**	Describe results from investigations of inconsistency. This may include such information as measures of model fit to compare consistency and inconsistency models, *P* values from statistical tests, or summary of inconsistency estimates from different parts of the treatment network.	-
Risk of bias across studies	22	Present results of any assessment of risk of bias across studies for the evidence base being studied.	10
Results of additional analyses	23	Give results of additional analyses, if done (e.g., sensitivity or subgroup analyses, meta-regression analyses, *alternative network geometries studied, alternative choice of prior distributions for Bayesian analyses,* and so forth).	10
**DISCUSSION**			
Summary of evidence	24	Summarize the main findings, including the strength of evidence for each main outcome; consider their relevance to key groups (e.g., healthcare providers, users, and policy-makers).	11
Limitations	25	Discuss limitations at study and outcome level (e.g., risk of bias), and at review level (e.g., incomplete retrieval of identified research, reporting bias). *Comment on the validity of the assumptions, such as transitivity and consistency. Comment on any concerns regarding network geometry (e.g., avoidance of certain comparisons).*	12-13

Conclusions	26	Provide a general interpretation of the results in the context of other evidence, and implications for future research.	13
**FUNDING**			
Funding	27	Describe sources of funding for the systematic review and other support (e.g., supply of data); role of funders for the systematic review. This should also include information regarding whether funding has been received from manufacturers of treatments in the network and/or whether some of the authors are content experts with professional conflicts of interest that could affect use of treatments in the network.	1

Supplement 2: Search Strategy used in each database

Source	Strategy
MEDLINE	(((("Temporomandibular Joint Disorders"[Mesh]) OR ((((temporomandibular) OR "Temporomandibular Joint"[Mesh])) AND (((((disorder*) OR dysfunction) OR osteoarthritis) OR "Osteoarthritis"[Mesh]) OR ((((derangement) OR displacement)) AND disc))))) AND ((((((((((((("Glucocorticoids"[Mesh]) OR Glucocorticoids) OR corticosteroid*) OR "Triamcinolone"[Mesh]) OR betamethasone) OR "Betamethasone"[Mesh]) OR dexamethasone) OR "Dexamethasone"[Mesh]) OR hydrocortisone) OR "Hydrocortisone"[Mesh]) OR methylprednisolone) OR "Methylprednisolone"[Mesh]) OR steroid) OR "Steroids"[Mesh]) OR triamcinolone)) AND ((clinical[Title/Abstract] AND trial[Title/Abstract]) OR clinical trials as topic[MeSH Terms] OR clinical trial[Publication Type] OR random*[Title/Abstract] OR random allocation[MeSH Terms] OR therapeutic use[MeSH Subheading])
EMBASE	(('temporomandibular joint'/exp OR 'temporomandibular joint' OR temporomandibular) AND (disorder* OR dysfunction OR 'osteoarthritis'/exp OR osteoarthritis) AND ('glucocorticoids'/exp OR glucocorticoids OR corticosteroid* OR 'triamcinolone'/exp OR triamcinolone OR 'betamethasone'/exp OR betamethasone OR 'dexamethasone'/exp OR dexamethasone OR 'methylprednisolone'/exp OR methylprednisolone OR 'hydrocortisone'/exp OR hydrocortisone OR 'steroids'/exp OR steroids)) AND (random OR 'clinical trial'/exp OR 'health care quality'/exp) AND [embase]/lim
SCOPUS	(TITLE-ABS-KEY ((((temporomandibular AND joint OR temporomandibular) AND (disorder* OR dysfunction OR osteoarthritis)) AND ((glucocorticoids OR corticosteroid* OR triamcinolone OR betamethasone OR dexamethasone OR methylprednisolone OR hydrocortisone OR steroids))))) AND ((TITLE-ABS-KEY (random*)) OR (TITLE-ABS-KEY (clinical AND trial)))
LILACS	(MH:"Temporomandibular Joint" OR "temporomandibular joint") AND (disorder$ OR dysfunction OR osteoarthritis OR MH:"Osteoarthritis" OR MH:"Temporomandibular Joint Disorders") AND (MH:"Glucocorticoid" OR MH:"Corticosteroids" OR MH:"Triamcinolone" MH:"Triamcinolone Acetonide" OR MH:"Betamethasone" OR MH:"Dexamethasone" OR MH:"Hydrocortisone" OR MH:"Methylprednisolone" OR MH:"Steroids")
COCHRANE CENTRAL	#1 MeSH descriptor: [Temporomandibular Joint] explode all trees<br>#2 MeSH descriptor: [Osteoarthritis] explode all trees<br>#3 MeSH descriptor: [Temporomandibular Joint Disorders] explode all trees<br>#4 #1 OR #2 OR #3<br>#5 MeSH descriptor: [Glucocorticoids] explode all trees<br>#6 MeSH descriptor: [Triamcinolone] explode all trees<br>#7 MeSH descriptor: [Betamethasone] explode all trees<br>#8 MeSH descriptor: [Dexamethasone] explode all trees<br>#9 MeSH descriptor: [Hydrocortisone] explode all trees<br>#10 MeSH descriptor: [Methylprednisolone] explode all trees<br>#11 MeSH descriptor: [Steroids] explode all trees<br>#12 MeSH descriptor: [Adrenal Cortex Hormones] explode all trees<br>#13 (corticosteroid):ti,ab,kw (Word variations have been searched)<br>#14 #5 OR #6 OR #7 OR #8 OR #9 OR #10 OR #11 OR #12 OR #13<br>#15 (clinical trial):ti,ab,kw (Word variations have been searched)<br>#16 (random*):ti,ab,kw (Word variations have been searched)<br>#17 #15 OR #16<br>#18 #4 AND #14 AND #17

Supplement 3: Characteristics of excluded studies

Study [ref]	Design	Country	N	Reasons for exclusion
Cömert-Kilic 2015 [1]	Randomized controlled trial	Turkey	38	Wrong outcomes (masticatory efficiency, progression of the osseous abnormalities in CBCT evaluations)
De Sousa 2020 [2]	Randomized controlled trial	Portugal	80	Wrong comparator (nocturnal bite splint)
Hammodi 2020 [3]	Not randomized controlled trial	Iraq	40	Wrong study design
Kopp 1981 [4]	Not randomized controlled trial	Sweden	15	Wrong study design
Kumar 2020 [5]	Randomized controlled trial	India	20	Wrong intervention (compare two techniques of arthrocentesis)
Pihut 2020 [6]	Not randomized controlled trial	Poland	100	Wrong study design<br>Wrong intervention (PRP and hyaluronic acid)
Schiffman 1996 [7]	Randomized controlled trial	USA	27	Wrong intervention (iontophoretic delivery of dexamethasone)
Schiffman 2007 [8]	Randomized controlled trial	USA	106	Wrong intervention (Medical management, Rehabilitation, Arthroscopy and Arthroplasty)
Wenneberg 1978 [9]	Not randomized controlled trial	Sweden	Not reported	Wrong study design
Wenneberg 1991 [10]	Cohort study	Sweden	16	Wrong study design

References

1. Cömert Kılıç, S.; Güngörmüş, M.; Sümbüllü, M.A. Long-term comparative clinical and CBTC evaluation of intra- articular Platelet-rich plasma (PRP) and Corticosteroid Injections for the temporomandibular Joints osteoarthritis treatment. *Osteoporos Int* **2015**, *26*, S359.
2. Sousa, B.M.; Lopez-Valverde, N.; Lopez-Valverde, A.; Caramelo, F.; Fraile, J.F.; Payo, J.H.; Rodrigues, M.J. Different Treatments in Patients with Temporomandibular Joint Disorders: A Comparative Randomized Study. *Medicina (Kaunas)* **2020**, *56*, doi:10.3390/medicina56030113.
3. Hammoodi, S.; Sami, W.A.; Abdulkareem, E. The Safety and Efficacy of Intra-Articular Platelet Rich Plasma Injections in Comparison to Intra-Articular Steroid Injections in Treatment of Temporomandibular Joint Disorders. *Int Medical J* **2020**, *27*, 789-792.
4. Kopp, S.; Wenneberg, B. Effects of occlusal treatment and intraarticular injections on temporomandibular joint pain and dysfunction. *Acta Odontol Scand* **1981**, *39*, 87-96, doi:10.3109/00016358109162265.

5. Kumar, A.; Gupta, A.; Ghosh, R.; Pandey, R.; Kumar, S. A Comparative Study Between Concentric Single-Needle Puncture Technique and Conventional 2-Needle Technique for Temporomandibular Joint Arthrocentesis Plus Corticosteroid Injections. *Craniomaxillofac Trauma Reconstr* **2020**, *13*, 99-104, doi:10.1177/1943387520911826.
6. Pihut, M.; Gala, A. The Application of Intra-Articulr Injections for Management of the Consequences of Disc Displacement without Reduction. *Int J Environ Res Public Health* **2020**, *17*, doi:10.3390/ijerph17134726.
7. Schiffman, E.L.; Braun, B.L.; Lindgren, B.R. Temporomandibular joint iontophoresis: a double-blind randomized clinical trial. *J Orofac Pain* **1996**, *10*, 157-165.
8. Schiffman, E.L.; Look, J.O.; Hodges, J.S.; Swift, J.Q.; Decker, K.L.; Hathaway, K.M.; Templeton, R.B.; Fricton, J.R. Randomized effectiveness study of four therapeutic strategies for TMJ closed lock. *J Dent Res* **2007**, *86*, 58-63, doi:10.1177/154405910708600109.
9. Wenneberg, B.; Kopp, S. Short term effect of intra-articular injections of a corticosteroid on temporomandibular joint pain and dysfunction. *Swed Dent J* **1978**, *2*, 189-196.
10. Wenneberg, B.; Kopp, S.; Gröndahl, H.G. Long-term effect of intra-articular injections of a glucocorticosteroid into the TMJ: a clinical and radiographic 8-year follow-up. *J Craniomandib Disord* **1991**, *5*, 11-18.

Supplement 4: Risk of Bias of the included studies

a) Pain outcome

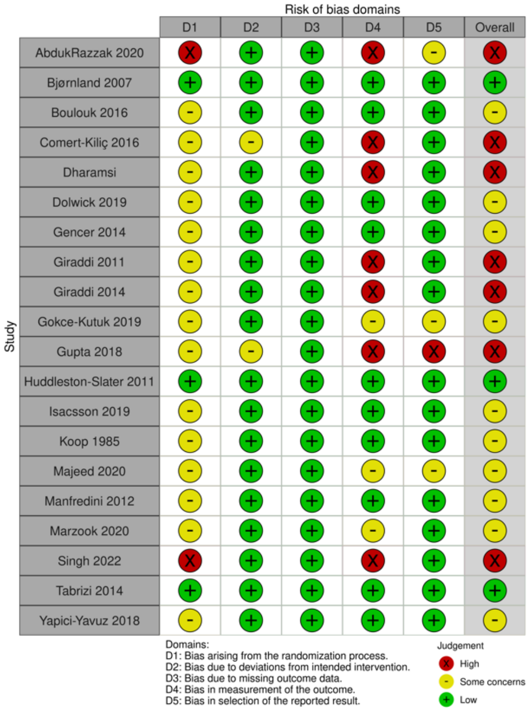

b) Range of Motion

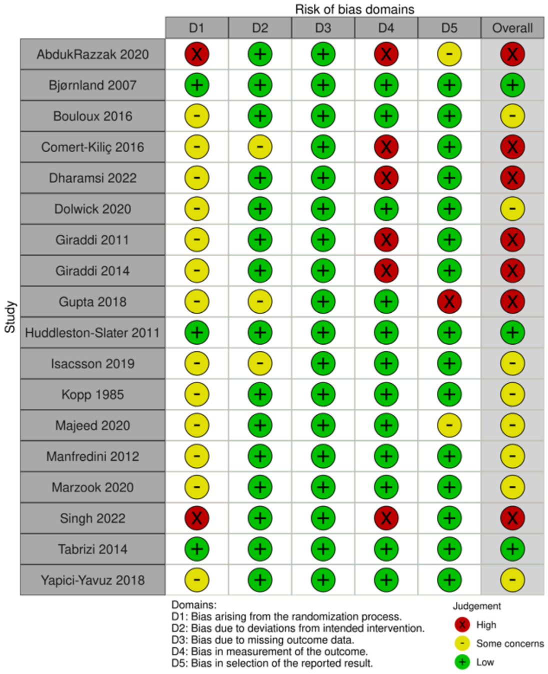

c) Adverse Event

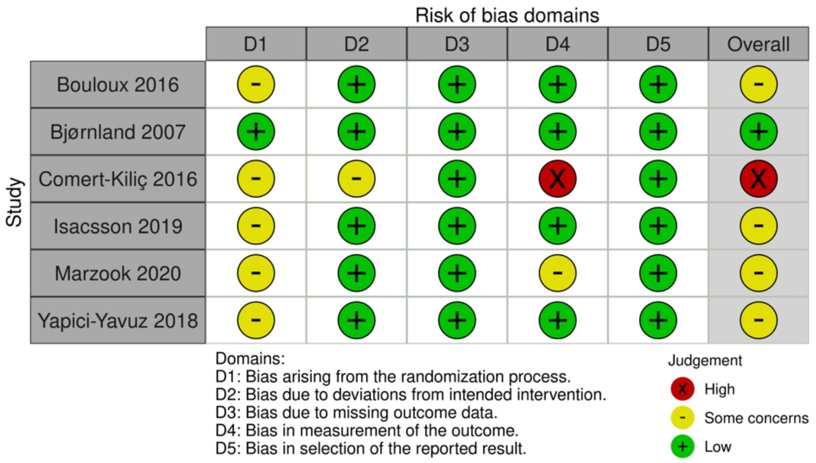

d) Quality of life

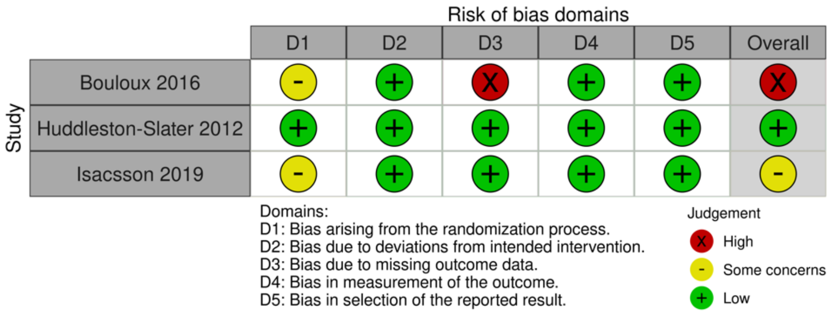

Supplement 5: Absolute effect estimates and certainty of evidence

5.1. Pain 1 month

COMPARISONS		DIRECT ESTIMATE				INDIRECT ESTIMATE				NETWORK ESTIMATE				
		ABSOLUTE ESTIMATE			GRADE	ABSOLUTE ESTIMATE			GRADE	ABSOLUTE ESTIMATE			GRADE	
TREATMENT 1	TREATMENT 2	Point estimate	CI lower limit	CI upper limit	**Final rating**	Point estimate	CI lower limit	CI upper limit	**Final rating**	Point estimate	CI lower limit	CI upper limit	**Final rating**	**Reasons for downgrading**
Arthrocentesis+Betamethasone	Arthrocentesis	NA	NA	NA	NA	-0.72	-2.44	0.99	VERY LOW	-0.72	-2.44	0.99	VERY LOW	Imprecision x2
Arthrocentesis+Dexamethasone	Arthrocentesis	-0.69	-1.50	0.13	MODERATE	NA	NA	NA	NA	-0.69	-1.50	0.13	LOW	Imprecision x2
Arthro+HA+Betamethasone	Arthrocentesis	NA	NA	NA	NA	-2.29	-4.07	-0.52	LOW	-2.29	-4.07	-0.52	LOW	Risk of Bias, Imprecision
Arthro+Methylprednisolone	Arthrocentesis	-0.37	-2.50	1.78	MODERATE	-3.88	-8.58	0.82	LOW	-1.13	-2.89	0.62	LOW	Imprecision x2
Arthrocentesis+Triamcinolone	Arthrocentesis	-0.96	-2.18	0.27	LOW	0.81	-1.70	3.32	VERY LOW	-0.61	-1.72	0.49	LOW	Imprecision x2
Betamethasone	Arthrocentesis	-3.8	-4.55	-3.05	LOW	11.64	8.58	14.69	LOW	-3.80	-4.55	-3.05	MODERATE	Imprecision
Triamcinolone	Arthrocentesis	NA	NA	NA	NA	-5.52	-6.56	-4.48	LOW	-5.52	-6.56	-4.48	LOW	Risk of Bias, Imprecision
Arthro+HA+Betamethasone	Arthro+Methylprednisolone	NA	NA	NA	NA	-1.16	-3.17	0.86	VERY LOW	-1.16	-3.17	0.86	VERY LOW	Risk of Bias, Imprecision x2
Arthro+Methylprednisolone	Arthrocentesis+Betamethasone	NA	NA	NA	NA	0.41	-1.55	2.37	VERY LOW	0.41	-1.55	2.37	VERY LOW	Risk of Bias, Imprecision x2
Arthro+Methylprednisolone	Arthrocentesis+Dexamethasone	NA	NA	NA	NA	0.45	-1.48	2.38	LOW	0.45	-1.48	2.38	LOW	Imprecision x2
Arthro+Methylprednisolone	Arthrocentesis+Triamcinolone	NA	NA	NA	NA	0.52	-1.20	2.24	LOW	0.52	-1.20	2.24	LOW	Imprecision x2
Arthro+Methylprednisolone	Betamethasone	NA	NA	NA	NA	-2.67	-4.57	-0.76	MODERATE	-2.67	-4.57	-0.76	MODERATE	Imprecision x2
Arthro+Methylprednisolone	Triamcinolone	NA	NA	NA	NA	-4.38	-6.42	-2.35	MODERATE	-4.38	-6.42	-2.35	LOW	Risk of Bias, Imprecision
Arthrocentesis+Betamethasone	Arthrocentesis+Dexamethasone	NA	NA	NA	NA	0.03	-1.86	1.93	VERY LOW	0.03	-1.86	1.93	VERY LOW	Risk of Bias, Imprecision x2
Arthrocentesis+Betamethasone	Arthro+HA+Betamethasone	-1.57	-2.03	-1.11	LOW	1.45	-52.33	55.24	VERY LOW	-1.57	-2.03	-1.11	LOW	Risk of Bias, Imprecision
Arthrocentesis+Betamethasone	Betamethasone	NA	NA	NA	NA	-3.08	-4.95	-1.21	LOW	-3.08	-4.95	-1.21	LOW	Risk of Bias, Imprecision
Arthrocentesis+Betamethasone	Arthrocentesis+Triamcinolone	NA	NA	NA	NA	0.11	-1.35	1.56	VERY LOW	0.11	-1.35	1.56	VERY LOW	Risk of Bias, Imprecision x2
Arthrocentesis+Betamethasone	Triamcinolone	NA	NA	NA	NA	-4.80	-6.80	-2.79	LOW	-4.80	-6.80	-2.79	LOW	Risk of Bias, Imprecision
Arthrocentesis+Dexamethasone	Arthro+HA+Betamethasone	NA	NA	NA	NA	-1.60	-3.56	0.35	VERY LOW	-1.60	-3.56	0.35	VERY LOW	Risk of Bias, Imprecision x2
Arthrocentesis+Dexamethasone	Arthrocentesis+Triamcinolone	NA	NA	NA	NA	0.07	-1.30	1.44	LOW	0.07	-1.30	1.44	LOW	Imprecision x2
Arthrocentesis+Dexamethasone	Betamethasone	NA	NA	NA	NA	-3.11	-4.22	-2.01	MODERATE	-3.11	-4.22	-2.01	MODERATE	Imprecision
Arthrocentesis+Dexamethasone	Triamcinolone	NA	NA	NA	NA	-4.83	-6.15	-3.51	LOW	-4.83	-6.15	-3.51	LOW	Risk of Bias, Imprecision
Arthro+HA+Betamethasone	Arthrocentesis+Triamcinolone	NA	NA	NA	NA	1.68	0.15	3.20	LOW	1.68	0.15	3.20	LOW	Risk of Bias, Imprecision
Arthro+HA+Betamethasone	Betamethasone	NA	NA	NA	NA	-1.51	-3.44	0.42	VERY LOW	-1.51	-3.44	0.42	VERY LOW	Risk of Bias, Imprecision x2
Arthro+HA+Betamethasone	Triamcinolone	NA	NA	NA	NA	-3.23	-5.28	-1.17	LOW	-3.23	-5.28	-1.17	LOW	Risk of Bias, Imprecision
Arthrocentesis+Triamcinolone	Betamethasone	NA	NA	NA	NA	-3.18	-4.52	-1.85	MODERATE	-3.18	-4.52	-1.85	MODERATE	Imprecision
Arthrocentesis+Triamcinolone	Triamcinolone	NA	NA	NA	NA	-4.90	-6.42	-3.40	LOW	-4.90	-6.42	-3.40	LOW	Risk of Bias, Imprecision
Betamethasone	Triamcinolone	NA	NA	NA	NA	-1.72	-2.82	-0.62	LOW	-1.72	-2.82	-0.62	LOW	Risk of Bias, Imprecision

5.2. Pain 3 months

COMPARISONS		DIRECT ESTIMATE				INDIRECT ESTIMATE				NETWORK ESTIMATE				
		ABSOLUTE ESTIMATE			GRADE	ABSOLUTE ESTIMATE			GRADE	ABSOLUTE ESTIMATE			GRADE	
TREATMENT 1	TREATMENT 2	Point estimate	CI lower limit	CI upper limit	Final rating	Point estimate	CI lower limit	CI upper limit	Final rating	Point estimate	CI lower limit	CI upper limit	**Final rating**	**Reasons for downgrading**
Arthrocentesis+Betamethasone	Arthrocentesis	NA	NA	NA	NA	-0.94	-2.90	1.02	VERY LOW	-0.94	-2.90	1.02	VERY LOW	Risk of Bias, Imprecision x2
Arthro+HA+Betamethasone	Arthrocentesis	NA	NA	NA	NA	-2.51	-4.51	-0.51	LOW	-2.51	-4.51	-0.51	LOW	Risk of Bias, Imprecision
Arthro+Methylprednisolone	Arthrocentesis	-0.09	-2.29	2.11	LOW	-7.90	-14.21	-1.60	MODERATE	-1.03	-3.07	1.01	LOW	Imprecision x2
Arthrocentesis+Triamcinolone	Arthrocentesis	-1.98	-3.81	-0.15	VERY LOW	1.96	-0.88	4.79	VERY LOW	-0.83	-2.36	0.71	LOW	Risk of Bias x2, Imprecision x2
Betamethasone	Arthrocentesis	-3.65	-4.36	-2.94	MODERATE	13.19	9.74	16.63	MODERATE	-2.74	-3.42	-2.06	MODERATE	Imprecision x2
Hydrocortisone	Arthrocentesis	NA	NA	NA	NA	-5.43	-6.50	-4.36	VERY LOW	-5.43	-6.50	-4.36	VERY LOW	Risk of Bias x2, Imprecision
Triamcinolone	Arthrocentesis	1.9	0.87	2.93	MODERATE	-6.52	-7.98	-5.05	MODERATE	-0.88	-1.72	-0.04	VERY LOW	Risk of Bias, Imprecision, Incoherence
Arthro+Methylprednisolone	Arthrocentesis+Betamethasone	NA	NA	NA	NA	0.09	-2.36	2.54	LOW	0.09	-2.36	2.54	LOW	Imprecision x2
Arthro+Methylprednisolone	Arthrocentesis+Triamcinolone	NA	NA	NA	NA	0.21	-2.02	2.43	LOW	0.21	-2.02	2.43	LOW	Imprecision x2
Arthro+Methylprednisolone	Arthro+HA+Betamethasone	NA	NA	NA	NA	-1.48	-3.96	0.10	VERY LOW	-1.48	-3.96	0.10	VERY LOW	Risk of Bias, Imprecision x2
Arthro+Methylprednisolone	Betamethasone	NA	NA	NA	NA	-1.71	-3.86	0.45	VERY LOW	-1.71	-3.86	0.45	VERY LOW	Imprecision x2
Arthro+Methylprednisolone	Hydrocortisone	NA	NA	NA	NA	-4.40	-6.70	-2.09	VERY LOW	-4.40	-6.70	-2.09	VERY LOW	Risk of Bias x2, Imprecision
Arthro+Methylprednisolone	Triamcinolone	NA	NA	NA	NA	0.15	-2.05	2.36	VERY LOW	0.15	-2.05	2.36	VERY LOW	Risk of Bias x2, Imprecision x2
Arthrocentesis+Betamethasone	Arthrocentesis+Triamcinolone	NA	NA	NA	NA	0.12	-1.33	1.56	VERY LOW	0.12	-1.33	1.56	VERY LOW	Risk of Bias x2, Imprecision x2
Arthrocentesis+Betamethasone	Arthro+HA+Betamethasone	-1.57	-1.97	-1.17	LOW	1.93	-31.46	35.33	VERY LOW	-1.57	-1.97	-1.17	LOW	Risk of Bias, Imprecision
Arthrocentesis+Betamethasone	Betamethasone	NA	NA	NA	NA	-1.80	-3.87	0.28	VERY LOW	-1.80	-3.87	0.28	VERY LOW	Risk of Bias, Imprecision
Arthrocentesis+Betamethasone	Hydrocortisone	NA	NA	NA	NA	-4.49	-6.72	-2.26	VERY LOW	-4.49	-6.72	-2.26	VERY LOW	Risk of Bias x2, Imprecision
Arthrocentesis+Betamethasone	Triamcinolone	NA	NA	NA	NA	0.06	-2.07	2.20	VERY LOW	0.06	-2.07	2.20	VERY LOW	Risk of Bias x2, Imprecision x2
Arthrocentesis+Triamcinolone	Arthro+HA+Betamethasone	NA	NA	NA	NA	-1.68	-3.18	-0.19	VERY LOW	-1.68	-3.18	-0.19	VERY LOW	Risk of Bias x2, Imprecision
Arthrocentesis+Triamcinolone	Betamethasone	NA	NA	NA	NA	-1.91	-3.59	-0.23	MODERATE	-1.91	-3.59	-0.23	MODERATE	Imprecision
Arthrocentesis+Triamcinolone	Hydrocortisone	NA	NA	NA	NA	-4.60	-6.48	-2.73	VERY LOW	-4.60	-6.48	-2.73	VERY LOW	Risk of Bias x2, Imprecision
Arthrocentesis+Triamcinolone	Triamcinolone	NA	NA	NA	NA	-0.05	-1.80	1.70	VERY LOW	-0.05	-1.80	1.70	VERY LOW	Risk of Bias x2, Imprecision x2
Arthro+HA+Betamethasone	Betamethasone	NA	NA	NA	NA	-0.23	-2.34	1.88	VERY LOW	-0.23	-2.34	1.88	VERY LOW	Risk of Bias, Imprecision x2
Arthro+HA+Betamethasone	Hydrocortisone	NA	NA	NA	NA	-2.92	-5.19	-0.65	VERY LOW	-2.92	-5.19	-0.65	VERY LOW	Risk of Bias x2, Imprecision
Arthro+HA+Betamethasone	Triamcinolone	NA	NA	NA	NA	1.63	-0.54	3.80	VERY LOW	1.63	-0.54	3.80	VERY LOW	Risk of Bias x2, Imprecision x2
Betamethasone	Hydrocortisone	NA	NA	NA	NA	-2.69	-3.74	-1.65	VERY LOW	-2.69	-3.74	-1.65	VERY LOW	Risk of Bias x2, Imprecision
Betamethasone	Triamcinolone	NA	NA	NA	NA	1.86	0.89	2.83	VERY LOW	1.86	0.89	2.83	VERY LOW	Risk of Bias x2, Imprecision
Hydrocortisone	Triamcinolone	NA	NA	NA	NA	4.55	3.41	5.70	VERY LOW	4.55	3.41	5.70	VERY LOW	Risk of Bias x2, Imprecision

5.3. Pain 6 months

COMPARISONS		DIRECT ESTIMATE				INDIRECT ESTIMATE				NETWORK ESTIMATE				
		ABSOLUTE ESTIMATE			GRADE	ABSOLUTE ESTIMATE			GRADE	ABSOLUTE ESTIMATE			GRADE	
TREATMENT 1	TREATMENT 2	Point estimate	CI lower limit	CI upper limit	**Final rating**	Point estimate	CI lower limit	CI upper limit	**Final rating**	Point estimate	CI lower limit	CI upper limit	**Final rating**	**Reasons for downgrading**
Arthrocentesis+Betamethasone	Arthrocentesis	NA	NA	NA	NA	0.33	-2.68	0.68	VERY LOW	0.33	-2.19	2.86	VERY LOW	Risk of Bias, Imprecision x2
Arthrocentesis+Dexamethasone	Arthrocentesis	-0.80	-1.57	-0.03	MODERATE	NA	NA	NA	NA	-0.80	-1.57	-0.03	MODERATE	Imprecision
Arthro+HA+Betamethasone	Arthrocentesis	NA	NA	NA	NA	-1.10	-3.69	1.49	VERY LOW	-1.10	-3.69	1.49	VERY LOW	Risk of Bias, Imprecision x2
Arthro+Methylprednisolone	Arthrocentesis	-1	-2.69	0.69	LOW	-0.38	-46.90	46.13	VERY LOW	-1	-2.68	0.68	LOW	Imprecision X2
Arthro+HA+Betamethasone	Arthrocentesis+Betamethasone	1.43	0.85	2.01	LOW	0.66	-48.39	49.70	VERY LOW	1.43	0.85	2.01	LOW	Risk of Bias, Imprecision
Arthro+HA+Betamethasone	Arthrocentesis+Dexamethasone	NA	NA	NA	NA	0.30	-2.41	3	VERY LOW	0.30	-2.41	3	VERY LOW	Risk of Bias, Imprecision x2
Arthro+HA+Betamethasone	Arthro+Methylprednisolone	NA	NA	NA	NA	0.98	-1.96	2.16	VERY LOW	0.98	-1.96	2.16	VERY LOW	Risk of Bias, Imprecision x2
Arthro+Methylprednisolone	Arthrocentesis+Betamethasone	NA	NA	NA	NA	1.33	-0.65	3.31	VERY LOW	1.33	-0.65	3.31	VERY LOW	Risk of Bias, Imprecision x2
Arthro+Methylprednisolone	Arthrocentesis+Dexamethasone	NA	NA	NA	NA	0.20	-1.65	2.05	LOW	0.20	-1.65	2.05	VERY LOW	Risk of Bias, Imprecision x2
Arthrocentesis+Betamethasone	Arthrocentesis+Dexamethasone	NA	NA	NA	NA	-1.13	-3.77	1.51	VERY LOW	-1.13	-3.77	1.51	VERY LOW	Risk of Bias, Imprecision x2

5.4. Range of Motion 1 month

COMPARISONS		DIRECT ESTIMATE				INDIRECT ESTIMATE				NETWORK ESTIMATE				
		ABSOLUTE ESTIMATE			GRADE	ABSOLUTE ESTIMATE			GRADE	ABSOLUTE ESTIMATE			GRADE	
TREATMENT 1	TREATMENT 2	Point estimate	CI lower limit	CI upper limit	Final rating	Point estimate	CI lower limit	CI upper limit	Final rating	Point estimate	CI lower limit	CI upper limit	**Final rating**	**Reasons for downgrading**
Arthrocentesis+Betamethasone	Arthrocentesis	1.33	-2.94	5.60	LOW	-3.70	-9.21	1.81	VERY LOW	-0.57	-3.90	2.75	VERY LOW	Imprecision x2
Arthrocentesis+Dexamethasone	Arthrocentesis	-0.5	-1.48	0.48	LOW	-0.25	-4.65	4.65		-0.33	-1.09	0.43	LOW	Imprecision x2
Arthro+HA+Betamethasone	Arthrocentesis	NA	NA	NA	NA	1.86	-2.15	5.86	VERY LOW	1.86	-2.15	5.86	VERY LOW	Risk of Bias, Imprecision x2
Arthro+Methylprednisolone	Arthrocentesis	-1.73	-6.99	3.53	LOW	2.24	-8.17	12.65	LOW	-0.77	-5.07	3.53	LOW	Imprecision x2
Arthrocentesis+Triamcinolone	Arthrocentesis	-0.54	-7.21	6.13	LOW	0.69	-6.12	7.50	VERY LOW	0.06	-4.70	4.83	LOW	Imprecision x2
HA+Triamcinolone	Arthrocentesis	-0.25	-4.73	4.23	LOW	NA	NA	NA	NA	-0.25	-4.73	4.23	LOW	Risk of Bias, Imprecision x2
Arthro+HA+Betamethasone	Arthrocentesis+Betamethasone	-2.43	-4.66	-0.20	MODERATE	-1.15	-259.26	256.97	VERY LOW	-2.43	-4.66	-0.20	MODERATE	Imprecision
Arthro+HA+Betamethasone	Arthrocentesis+Dexamethasone	NA	NA	NA	NA	-2.19	-6.26	1.89	VERY LOW	-2.19	-6.26	1.89	VERY LOW	Risk of Bias, Imprecision x2
Arthro+HA+Betamethasone	Arthrocentesis+Triamcinolone	NA	NA	NA	NA	-1.79	-7.46	3.88	VERY LOW	-1.79	-7.46	3.88	VERY LOW	Risk of Bias, Imprecision x2
Arthro+HA+Betamethasone	Arthro+Methylprednisolone	NA	NA	NA	NA	-2.62	-7.72	2.48	VERY LOW	-2.62	-7.72	2.48	VERY LOW	Risk of Bias, Imprecision x2
Arthro+HA+Betamethasone	HA+Triamcinolone	NA	NA	NA	NA	-2.11	-8.12	3.91	VERY LOW	-2.11	-8.12	3.91	VERY LOW	Risk of Bias, Imprecision x2
Arthro+Methylprednisolone	Arthrocentesis+Betamethasone	NA	NA	NA	NA	0.19	-4.39	4.78	LOW	0.19	-4.39	4.78	LOW	Imprecision x2
Arthro+Methylprednisolone	Arthrocentesis+Dexamethasone	NA	NA	NA	NA	0.44	-3.93	4.80	LOW	0.44	-3.93	4.80	LOW	Imprecision x2
Arthro+Methylprednisolone	Arthrocentesis+Triamcinolone	NA	NA	NA	NA	0.83	-5.06	6.73	LOW	0.83	-5.06	6.73	LOW	Imprecision x2
Arthro+Methylprednisolone	HA+Triamcinolone	NA	NA	NA	NA	0.52	-5.69	6.73	VERY LOW	0.52	-5.69	6.73	VERY LOW	Risk of Bias, Imprecision x2
Arthrocentesis+Betamethasone	Arthrocentesis+Dexamethasone	NA	NA	NA	NA	0.24	-3.17	3.66	LOW	0.24	-3.17	3.66	LOW	Imprecision x2
Arthrocentesis+Betamethasone	Arthrocentesis+Triamcinolone	NA	NA	NA	NA	0.64	-4.58	5.85	LOW	0.64	-4.58	5.85	LOW	Imprecision x2
Arthrocentesis+Betamethasone	HA+Triamcinolone	NA	NA	NA	NA	0.32	-5.26	5.91	LOW	0.32	-5.26	5.91	LOW	Imprecision x2
Arthrocentesis+Dexamethasone	Arthrocentesis+Triamcinolone	NA	NA	NA	NA	0.39	-4.43	5.22	LOW	0.39	-4.43	5.22	LOW	Imprecision x2
Arthrocentesis+Dexamethasone	HA+Triamcinolone	NA	NA	NA	NA	0.08	-4.47	4.63	LOW	0.08	-4.47	4.63	LOW	Imprecision x2
Arthrocentesis+Triamcinolone	HA+Triamcinolone	NA	NA	NA	NA	-0.31	-6.86	6.23	LOW	-0.31	-6.86	6.23	LOW	Imprecision x2

5.5. Range of Motion 3 months

COMPARISONS		DIRECT ESTIMATE				INDIRECT ESTIMATE				NETWORK ESTIMATE				
		ABSOLUTE ESTIMATE			GRADE	ABSOLUTE ESTIMATE			GRADE	ABSOLUTE ESTIMATE			GRADE	
TREATMENT 1	TREATMENT 2	Point estimate	CI lower limit	CI upper limit	Final rating	Point estimate	CI lower limit	CI upper limit	Final rating	Point estimate	CI lower limit	CI upper limit	**Final rating**	**Reasons for downgrading**
Arthrocentesis+Betamethasone	Arthrocentesis	-1.92	-7.06	3.22	LOW	-2.44	-7.83	2.95	VERY LOW	-2.17	-5.79	1.45	LOW	Imprecision x2
Arthro+HA+Betamethasone	Arthrocentesis	NA	NA	NA	NA	1.11	-2.84	5.06	VERY LOW	1.11	-2.84	5.06	VERY LOW	Risk of Bias, Imprecision x2
Arthro+Methylprednisolone	Arthrocentesis	0.46	-4.16	5.08	LOW	-1.73	-12.89	9.43	VERY LOW	0.12	-4.10	4.34	LOW	Imprecision x2
Arthrocentesis+Triamcinolone	Arthrocentesis	-0.71	-8.63	7.21	LOW	1.71	-5.41	8.83	VERY LOW	0.63	-4.67	5.92	VERY LOW	Risk of Bias, Imprecision x2
HA+Triamcinolone	Arthrocentesis	0.5	-4.04	5.04	LOW	NA	NA	NA	NA	0.5	-4.04	5.04	LOW	Imprecision x2
Triamcinolone	Arthrocentesis	-4.70	-10.77	1.37	VERY LOW	NA	NA	NA	NA	-4.70	-10.77	1.37	VERY LOW	Risk of Bias, Imprecision x2
HA+Triamcinolone	Arthrocentesis+Betamethasone	NA	NA	NA	NA	-2.67	-8.48	3.14	LOW	-2.67	-8.48	3.14	LOW	Imprecision x2
HA+Triamcinolone	Arthrocentesis+Triamcinolone	NA	NA	NA	NA	0.13	-6.84	7.10	LOW	0.13	-6.84	7.10	LOW	Imprecision x2
HA+Triamcinolone	Arthro+HA+Betamethasone	NA	NA	NA	NA	0.61	-5.40	6.63	VERY LOW	0.61	-5.40	6.63	VERY LOW	Risk of Bias, Imprecision x2
HA+Triamcinolone	Triamcinolone	NA	NA	NA	NA	-5.20	-12.78	2.38	VERY LOW	-5.20	-12.78	2.38	VERY LOW	Risk of Bias x2, Imprecision x2
HA+Triamcinolone	Arthro+Methylprednisolone	NA	NA	NA	NA	-0.38	-6.57	5.82	LOW	-0.38	-6.57	5.82	LOW	Imprecision x2
Arthro+Methylprednisolone	Arthrocentesis+Betamethasone	NA	NA	NA	NA	-2.29	-7.21	2.63	LOW	-2.29	-7.21	2.63	LOW	Imprecision x2
Arthro+Methylprednisolone	Arthrocentesis+Triamcinolone	NA	NA	NA	NA	0.51	-5.87	6.88	LOW	0.51	-5.87	6.88	LOW	Imprecision x2
Arthro+Methylprednisolone	Arthro+HA+Betamethasone	NA	NA	NA	NA	0.99	-4.18	6.15	VERY LOW	0.99	-4.18	6.15	VERY LOW	Risk of Bias, Imprecision x2
Arthro+Methylprednisolone	Triamcinolone	NA	NA	NA	NA	-4.82	-12.21	2.57	VERY LOW	-4.82	-12.21	2.57	VERY LOW	Risk of Bias x2, Imprecision x2
Arthrocentesis+Betamethasone	Arthrocentesis+Triamcinolone	NA	NA	NA	NA	2.80	-2.94	8.54	VERY LOW	2.80	-2.94	8.54	VERY LOW	Risk of Bias, Imprecision x2
Arthrocentesis+Betamethasone	Arthro+HA+Betamethasone	3.28	1.70	4.86	LOW	4.34	-172.64	181.32	VERY LOW	3.28	1.70	4.86	VERY LOW	Risk of Bias, Imprecision x2
Arthrocentesis+Betamethasone	Triamcinolone	NA	NA	NA	NA	-2.53	-9.60	4.54	VERY LOW	-2.53	-9.60	4.54	VERY LOW	Risk of Bias x2, Imprecision x2
Arthrocentesis+Triamcinolone	Arthro+HA+Betamethasone	NA	NA	NA	NA	0.48	-5.47	6.43	VERY LOW	0.48	-5.47	6.43	VERY LOW	Risk of Bias, Imprecision x2
Arthrocentesis+Triamcinolone	Triamcinolone	NA	NA	NA	NA	-5.33	-13.38	2.72	VERY LOW	-5.33	-13.38	2.72	VERY LOW	Risk of Bias x2, Imprecision x2
Arthro+HA+Betamethasone	Triamcinolone	NA	NA	NA	NA	-5.81	-13.05	1.43	VERY LOW	-5.81	-13.05	1.43	VERY LOW	Risk of Bias x2, Imprecision x2

5.6. Range of Motion 6 months

COMPARISONS		DIRECT ESTIMATE				INDIRECT ESTIMATE				NETWORK ESTIMATE				
		ABSOLUTE ESTIMATE			GRADE	ABSOLUTE ESTIMATE			GRADE	ABSOLUTE ESTIMATE			GRADE	
TREATMENT 1	TREATMENT 2	Point estimate	CI lower limit	CI upper limit	Final rating	Point estimate	CI lower limit	CI upper limit	Final rating	Point estimate	CI lower limit	CI upper limit	**Final rating**	**Reasons for downgrading**
Arthrocentesis+Betamethasone	Arthrocentesis	NA	NA	NA	NA	-3.92	-10.07	2.23	VERY LOW	-3.92	-10.07	2.23	VERY LOW	Risk of Bias, Imprecision x2
Arthrocentesis+Dexamethasone	Arthrocentesis	-0.27	-1.14	0.60	LOW	NA	NA	NA	NA	-0.27	-1.14	0.60	LOW	Imprecision x2
Arthro+HA+Betamethasone	Arthrocentesis	NA	NA	NA	NA	-0.20	-6.54	6.14	VERY LOW	-0.20	-6.54	6.14	VERY LOW	Risk of Bias, Imprecision x2
Arthro+Methylprednisolone	Arthrocentesis	2.19	-3.32	7.70	LOW	6.56	-153.57	-166.68	VERY LOW	2.20	-3.31	7.70	LOW	Imprecision x2
Arthro+HA+Betamethasone	Arthrocentesis+Betamethasone	-3.72	-5.28	-2.16	LOW	7.86	-166.26	188.97	VERY LOW	-3.72	-5.28	-2.16	LOW	Risk of Bias, Imprecision
Arthro+HA+Betamethasone	Arthrocentesis+Dexamethasone	NA	NA	NA	NA	-0.07	-6.47	6.33	VERY LOW	-0.07	-6.47	6.33	VERY LOW	Risk of Bias, Imprecision x2
Arthro+HA+Betamethasone	Arthro+Methylprednisolone	NA	NA	NA	NA	2.39	-3.35	8.14	VERY LOW	2.39	-3.35	8.14	VERY LOW	Risk of Bias, Imprecision x2
Arthro+Methylprednisolone	Arthrocentesis+Betamethasone	NA	NA	NA	NA	-6.11	-11.64	-0.59	LOW	-6.11	-11.64	-0.59	LOW	Risk of Bias, Imprecision x2
Arthro+Methylprednisolone	Arthrocentesis+Dexamethasone	NA	NA	NA	NA	-2.47	-8.04	3.10	VERY LOW	-2.47	-8.04	3.10	VERY LOW	Risk of Bias, Imprecision
Arthrocentesis+Betamethasone	Arthrocentesis+Dexamethasone	NA	NA	NA	NA	3.65	'-2.56	9.86	VERY LOW	3.65	'-2.56	9.86	VERY LOW	Risk of Bias, Imprecision x2

Reference supplements 4 and 5

1. AbdulRazzak, N.J.; Sadiq, J.A.; Jiboon, A.T. Arthrocentesis versus glucocorticosteroid injection for internal derangement of temporomandibular joint. *Oral Maxillofac Surg* **2021**, *25*, 191-197, doi:10.1007/s10006-020-00901-3.
2. Bjørnland, T.; Gjaerum, A.A.; Møystad, A. Osteoarthritis of the temporomandibular joint: an evaluation of the effects and complications of corticosteroid injection compared with injection with sodium hyaluronate. *J Oral Rehabil* **2007**, *34*, 583-589, doi:10.1111/j.1365-2842.2007.01759.x.
3. Møystad, A.; Mork-Knutsen, B.B.; Bjørnland, T. Injection of sodium hyaluronate compared to a corticosteroid in the treatment of patients with temporomandibular joint osteoarthritis: a CT evaluation. *Oral Surg Oral Med Oral Pathol Oral Radiol Endod* **2008**, *105*, e53-60, doi:10.1016/j.tripleo.2007.08.024.
4. Moystad, A.; Bjornland, T.; Mork-Knutsen, B.B.; Gjaerum, A.A. Injection of sodium hyaluronate compared with a corticosteroid in the treatment of patients with temporomandibular joint osteoarthritis: clinical effects and computed tomography evaluation of osseous changes. *Oral Surgery* **2008**, *1*, 88-95.
5. Bouloux, G.F.; Chou, J.; Krishnan, D.; Aghaloo, T.; Kahenasa, N.; Smith, J.A.; Giannakopoulos, H. Is Hyaluronic Acid or Corticosteroid Superior to Lactated Ringer Solution in the Short Term for Improving Function and Quality of Life After Arthrocentesis? Part 2. *J Oral Maxillofac Surg* **2017**, *75*, 63-72, doi:10.1016/j.joms.2016.08.008.
6. Bouloux, G.F.; Chou, J.; Krishnan, D.; Aghaloo, T.; Kahenasa, N.; Smith, J.A.; Giannakopoulos, H. Is Hyaluronic Acid or Corticosteroid Superior to Lactated Ringer Solution in the Short-Term Reduction of Temporomandibular Joint Pain After Arthrocentesis? Part 1. *J Oral Maxillofac Surg* **2017**, *75*, 52-62, doi:10.1016/j.joms.2016.08.006.
7. Cömert Kiliç, S. Does Injection of Corticosteroid After Arthrocentesis Improve Outcomes of Temporomandibular Joint Osteoarthritis? A Randomized Clinical Trial. *J Oral Maxillofac Surg* **2016**, *74*, 2151-2158, doi:10.1016/j.joms.2016.05.027.
8. Dharamsi, R.; Nilesh, K.; Mouneshkumar, C.D.; Patil, P. Use of Sodium Hyaluronate and Triamcinolone Acetonide Following Arthrocentesis in Treatment of Internal Derangement of Temporomandibular Joint: A Prospective Randomized Comparative Study. *J Maxillofac Oral Surg* **2022**, doi:https://doi.org/10.1007/s12663-022-01804-4.
9. Dolwick, M.F.; Diaz, D.; Freburg-Hoffmeister, D.L.; Widmer, C.G. A Randomized, Double-Blind, Placebo-Controlled Study of the Efficacy of Steroid Supplementation After Temporomandibular Joint Arthrocentesis. *J Oral Maxillofac Surg* **2020**, *78*, 1088-1099, doi:10.1016/j.joms.2020.02.022.
10. Diaz, D.; Dolwick, M.F.; Freburg-Hoffmeister, D.L.; Widmer, C.G. Double-Blind, Randomized, Placebo Controlled Clinical Trial Examining the Efficacy of Steroid Supplementation after TMJ Arthrocentesis. *AAOMS* **2019**, *77*, E50-E51.

11. Gencer, Z.K.; Özkiriş, M.; Okur, A.; Korkmaz, M.; Saydam, L. A comparative study on the impact of intra-articular injections of hyaluronic acid, tenoxicam and betametazon on the relief of temporomandibular joint disorder complaints. *J Craniomaxillofac Surg* **2014**, *42*, 1117-1121, doi:10.1016/j.jcms.2014.01.041.
12. Giraddi, G.B.; Siddaraju, A.; Kumar, B.; Singh, C. Internal derangement of temporomandibular joint: an evaluation of effect of corticosteroid injection compared with injection of sodium hyaluronate after arthrocentesis. *J Maxillofac Oral Surg* **2012**, *11*, 258-263, doi:10.1007/s12663-011-0324-8.
13. Giraddi, G.B.; Siddaraju, A.; Kumar, A.; Jain, T. Comparison Between Betamethasone and Sodium Hyaluronate Combination with Betamethasone Alone After Arthrocentesis in the Treatment of Internal Derangement of TMJ-Using Single Puncture Technique: A Preliminary Study. *J Maxillofac Oral Surg* **2015**, *14*, 403-409, doi:10.1007/s12663-014-0626-8.
14. Gokçe Kutuk, S.; Gökçe, G.; Arslan, M.; Özkan, Y.; Kütük, M.; Kursat Arikan, O. Clinical and Radiological Comparison of Effects of Platelet-Rich Plasma, Hyaluronic Acid, and Corticosteroid Injections on Temporomandibular Joint Osteoarthritis. *J Craniofac Surg* **2019**, *30*, 1144-1148, doi:10.1097/scs.0000000000005211.
15. Gupta, S.; Sharma, A.K.; Purohit, J.; Goyal, R.; Malviya, Y.; Jain, S. Comparison between intra-articular platelet-rich plasma injection versus hydrocortisone with local anesthetic injections in temporomandibular disorders: A double-blind study. *Natl J Maxillofac Surg* **2018**, *9*, 205-208, doi:10.4103/njms.NJMS_69_16.
16. Huddleston Slater, J.J.; Vos, L.M.; Stroy, L.P.; Stegenga, B. Randomized trial on the effectiveness of dexamethasone in TMJ arthrocentesis. *J Dent Res* **2012**, *91*, 173-178, doi:10.1177/0022034511431260.
17. Isacsson, G.; Schumann, M.; Nohlert, E.; Mejersjo, C.; Tegelberg, A. Pain relief following a single-dose intra-articular injection of methylprednisolone in the temporomandibular joint arthralgia-A multicentre randomised controlled trial. *J Oral Rehabil* **2019**, *46*, 5-13, doi:10.1111/joor.12718.
18. Kopp, S.; Wenneberg, B.; Haraldson, T.; Carlsson, G.E. The short-term effect of intra-articular injections of sodium hyaluronate and corticosteroid on temporomandibular joint pain and dysfunction. *J Oral Maxillofac Surg* **1985**, *43*, 429-435, doi:10.1016/s0278-2391(85)80050-1.
19. Manfredini, D.; Rancitelli, D.; Ferronato, G.; Guarda-Nardini, L. Arthrocentesis with or without additional drugs in temporomandibular joint inflammatory-degenerative disease: comparison of six treatment protocols*. *J Oral Rehabil* **2012**, *39*, 245-251, doi:10.1111/j.1365-2842.2011.02265.x.
20. Majeed, S.; Hassan, M.; Ali, Z. Assessment the Efficacy of Arthrocentesis with Corticosteroid and Arthrocentesis with Sodium Hyaluronate in Treatment Temporomandibular Joint Disorders: A Comparative Study. *Indian J Med Forensic Med Toxicol* **2020**, *14*, 361-366.
21. Marzook, H.A.M.; Abdel Razek, A.A.; Yousef, E.A.; Attia, A. Intra-articular injection of a mixture of hyaluronic acid and corticosteroid versus arthrocentesis in TMJ internal derangement. *J Stomatol Oral Maxillofac Surg* **2020**, *121*, 30-34, doi:10.1016/j.jormas.2019.05.003.

22. Singh, S.U.; Prasad, R.B.; Punga, R.; Datta, R.; Singh, N. A comparison of the outcomes following intra-articular steroid injection alone or arthrocentesis alone in the management of internal derangement of the temporomandibular joint. *Natl J Maxillofac Surg* **2022**, *13*, S80-S84, doi:10.4103/njms.njms_291_21.
23. Tabrizi, R.; Karagah, T.; Arabion, H.; Soleimanpour, M.R.; Soleimanpour, M. Outcomes of arthrocentesis for the treatment of internal derangement pain: with or without corticosteroids? *J Craniofac Surg* **2014**, *25*, e571-575, doi:10.1097/scs.0000000000001168.
24. Yapici-Yavuz, G.; Şimşek-Kaya, G.; Oğul, H. A comparison of the effects of Methylprednisolone Acetate, Sodium Hyaluronate and Tenoxicam in the treatment of non-reducing disc displacement of the temporomandibular joint. *Med Oral Patol Oral Cir Bucal* **2018**, *23*, e351-e358, doi:10.4317/medoral.22237.
